# Current Management of Pulmonary Hydatid Cyst

**DOI:** 10.5152/eurasianjmed.2025.24761

**Published:** 2025-02-03

**Authors:** Yener Aydın, Ali Bilal Ulaş, Ayşenur Dostbil

**Affiliations:** 1 Department of Thoracic Surgery, Atatürk University Faculty of Medicine, Erzurum, Türkiye; 2 Department of Anesthesiology, Atatürk University Faculty of Medicine, Erzurum, Türkiye

**Keywords:** Albendazole, cystotomy and capitonnage, hydatid cyst, lung, surgery

## Abstract

Hydatid disease is a zoonotic infection caused by the larval stage of the *Echinococcus granulosus* worm, commonly found in developing countries. The lungs represent the second most commonly affected organ in both children and adults. The disease is more common in children than in adults, and the growth of hydatid cysts is more rapid in children than in adults. Diagnosing uncomplicated cases of hydatid cysts is generally straightforward clinically and radiologically. However, difficulties may arise in diagnosing complicated cysts. Surgery is the definitive treatment for pulmonary hydatid cysts. The surgical approach is contingent upon a number of factors, including the dimensions of the cyst, the integrity of its structure, whether it is solitary or multiple, unilateral or bilateral, and the extent of lung parenchyma destruction. In contrast to the liver, surgical treatment is promptly planned upon the diagnosis of a pulmonary hydatid cyst. The most effective surgical method is cystotomy and capitonnage while maintaining the integrity of the lung parenchyma to the greatest extent possible. Albendazole is the preferred medical treatment, but it is not recommended for intact cysts during the preoperative period due to its potential to weaken the cyst wall and cause rupture. Albendazole is administered to prevent postoperative recurrence and for treatment in cases where surgery is not feasible.

**Main Points**

- Pulmonary hydatid cysts still commonly occur in developing countries.
- In children, lung involvement is more common and hydatid cyst growth is faster than in adults.
- Surgical treatment is the gold standard and depends on a number of factors, including the size of the cyst, rupture, solitary or multiple, unilateral or bilateral, and the degree of lung parenchymal destruction.
- Surgical management should include cystotomy and capitonnage with preservation of the lung parenchyma as much as possible.
- Modified capitonnage technique for giant hydatid cysts is effective in reducing complication rates.

## Introduction

Hydatid cyst disease is a zoonotic illness caused by the *E. granulosus* tapeworm. The liver is the most commonly affected organ, accounting for approximately 80% of cases. The lungs are the second most commonly affected organ, accounting for around 23% of all cases of hydatid cysts.^1^ This disease remains a significant public health concern in countries where definitive hosts, such as dogs, have close contact with humans and animals. It is pretty common for uncomplicated hydatid cysts to be diagnosed through chest X-rays, often as a result of the scan.^2^ Treatment methods for pulmonary hydatid cysts vary according to clinical practices and include medical therapy and surgical techniques. This review examines these treatment methods.

## Epidemiology

Hydatid cyst disease is a widespread global issue. However, due to insufficient data in many countries, a comprehensive global assessment of the current situation is not possible. The prevalence and incidence of the disease vary significantly between countries and regions. Endemic rural areas have reported prevalence rates of 2%-6% or higher.^3^ Parasite prevalence is high in Mediterranean countries, the Russian Federation, Turkic Republics, Australia, North and East Africa, and South America. Prevalence increases with age, and incidence is 2-6 times higher in rural areas. Additionally, it is slightly higher in women compared to men.^4,5^ However, a large series study comprising 3090 cases conducted in a single center found that the disease was most commonly seen in the second decade of life, with cases observed in every age group. It was approximately 4.6 times more prevalent in rural areas where animal husbandry is common practice. When comparing gender, it has been observed that around 60% of adult cases are female, while approximately 55% of children affected are male.^1^

## Life Cycle of *Echinonococcus granulosus*

Echinococcus has a definitive host, usually dogs, and an intermediate host, which is typically goats, sheep, camels, pigs, and cattle. Adult *E. granulosus* tapeworms are typically found in canids, specifically dogs. Humans are incidental hosts and do not contribute to the transmission cycle. Completion of the life cycle requires 2 mammalian species. It can be concluded that there is no transmission of *E. granulosus* from human to human.^6^

The adult tapeworm resides within the small intestine of the definitive host. Thousands of worms can infect the definitive host. The *E. granulosus* worm is typically 2 to 7 mm in length and comprises a scolex with suckers and hooks, as well as at least 3 proglottid segments. The tapeworm is composed of proglottid segments containing both male and female reproductive organs and embryos, which can produce parasite eggs measuring 30-40 microns. Each adult worm has the ability to produce thousands of eggs daily. The eggs are excreted into the environment via the feces of the definitive host, making them infectious to susceptible intermediate hosts and incidental human hosts. Due to their high resistance, the eggs can remain infective for up to a year in a moist environment with low temperatures. When an intermediate host ingests the egg orally, oncospheres are released. Once the larvae have penetrated the intestinal mucosa, they enter the bloodstream and/or lymphatic system. They then migrate to the liver or other internal organs. After several days, a fluid-filled cyst begins to develop, and multiple layers subsequently form a metacestode, known as a hydatid cyst.^7,8^

## Pulmonary Involvement

The eggs enter the intermediate host’s body and can penetrate the gastric acid barrier. They continue to develop in the small intestine. After the egg’s protective shell dissolves due to digestive enzymes, the liberated embryo attaches to the intestinal mucosa with its hooks. It then passes from the walls of the jejunum and ileum to the portal vein or periduodenal and perigastric lymphatics. The hydatid cyst typically develops in the liver as the oncosphere generally cannot pass through the liver sinusoids. If the larvae do manage to pass through the sinusoids, they can reach the liver vein, inferior vena cava, right heart, and pulmonary arteries, ultimately reaching the lungs. In rare cases, they may be transported via the thoracic duct or pass from the iliac veins to the inferior vena cava without passing through the liver, resulting in pulmonary involvement.^9^ Although publications often mention that pulmonary hydatid cysts are the most common in children, our extensive series at our clinic has shown that the liver is the organ most frequently affected.^10^ However, pulmonary involvement in children is more than twice as common as in adults.^1^

## Growth of Pulmonary Hydatid Cyst

The growth rate of hydatid cysts is contingent upon the degree of softness of the organ in question and the elasticity of the surrounding tissue. According to the literature, pulmonary hydatid cysts may grow faster than hepatic ones due to the lung’s softer nature and negative pleural pressure. Moreover, it has been observed that hydatid cysts may grow faster in children than in adults due to the more elastic lung tissues.^6,9^ However, demonstrating this growth through a prospective randomized study is not feasible due to ethical reasons. Therefore, these statements represent an analysis of surgeons’ clinical observations. A recent study of a large series of patients from 2 centers reported a doubling time of 73 days for pulmonary hydatid cysts and 173 days for hepatic hydatid cysts. In children, the doubling time for pulmonary and hepatic hydatid cysts was found to be 61 days and 111 days, respectively.^11^

## Clinical Findings

The initial stages of pulmonary hydatid cysts are frequently asymptomatic. The symptoms and signs experienced by patients are contingent upon the location and size of the cyst. Small, intact cysts are typically diagnosed incidentally and do not cause significant symptoms.^12,13^ The literature commonly reports cough (61%), chest pain (44%), dyspnea (25%), expectoration of sputum (17%), and hemoptysis (12%) as the most frequently encountered symptoms in cases of pulmonary hydatid cysts. Less frequently, patients may experience fever, fatigue, nausea, and vomiting. In approximately 7% of cases, expectoration of cyst fluid or membranes (hydatoptysis) is the definitive symptom, indicating that the cyst has perforated and opened into the bronchus.^1^ Following perforation, the patient’s general condition may deteriorate, leading to dyspnea and chest pain. Acute hypersensitivity reactions, including fever and anaphylaxis, are the most definitive signs of a ruptured cyst. Anaphylaxis can rarely result in death. Peripheral cysts may cause chest pain due to pleural irritation, as well as shoulder and abdominal pain due to diaphragmatic irritation. If the cyst ruptures into the pleura, it can lead to pneumothorax, pleural effusion, empyema, and associated symptoms and signs.^6,9^

## Diagnosis

**Figure 1.**
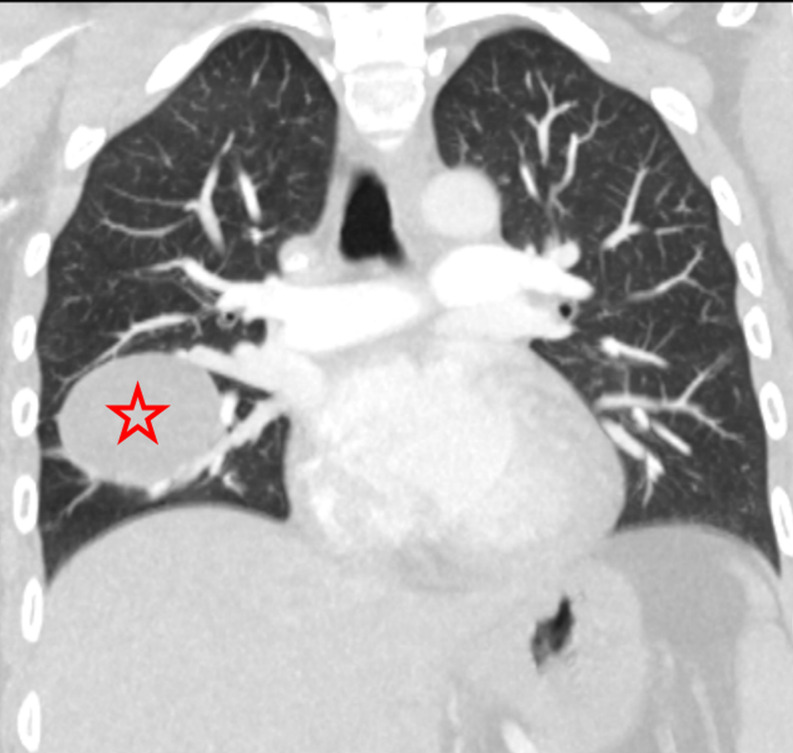
Coronal section of chest computed tomography (CT) with lung window in a 32-year-old female patient showing an intact hydatid cyst (asterisk).

**Figure 2.**
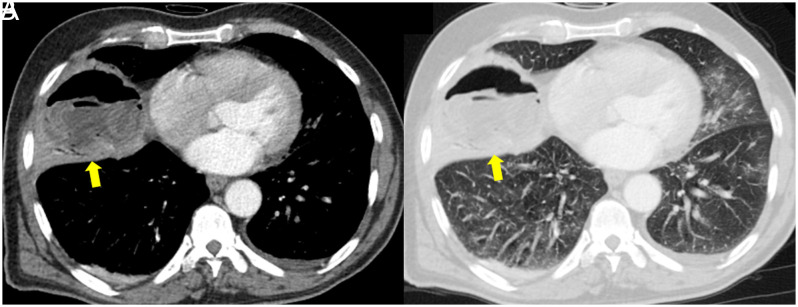
Axial section of chest CT in a 68-year-old male patient showing a complicated hydatid cyst in the right middle lobe of the lung as seen in the mediastinal (A) and lung (B) windows (arrow).

**Figure 3.**
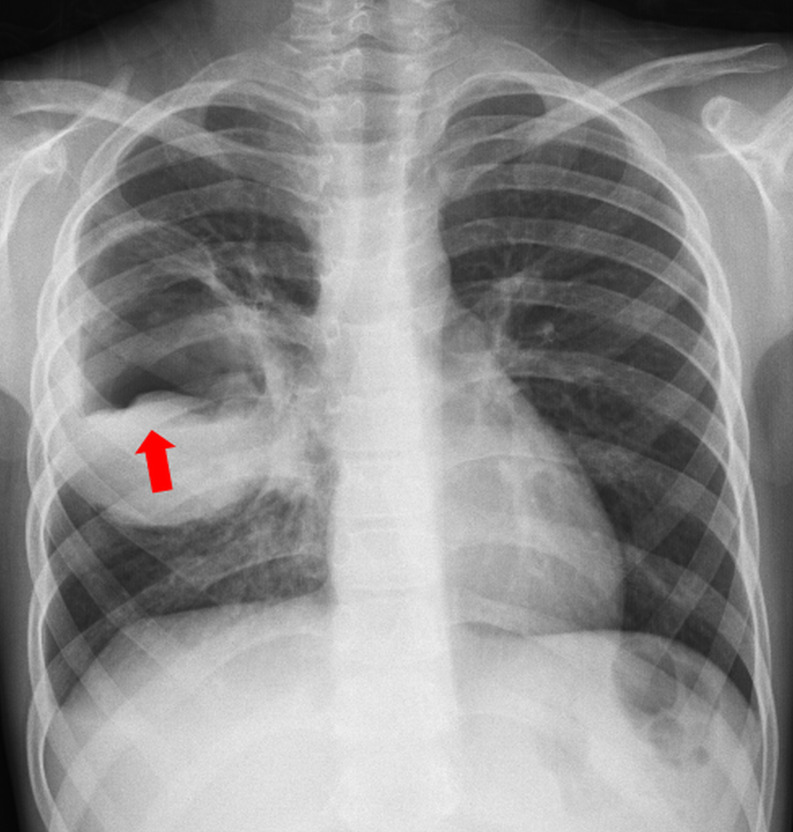
A 12-year-old male patient with a ruptured hydatid cyst in the right upper lobe causing a water-lily sign (arrow).

**Figure 4.**
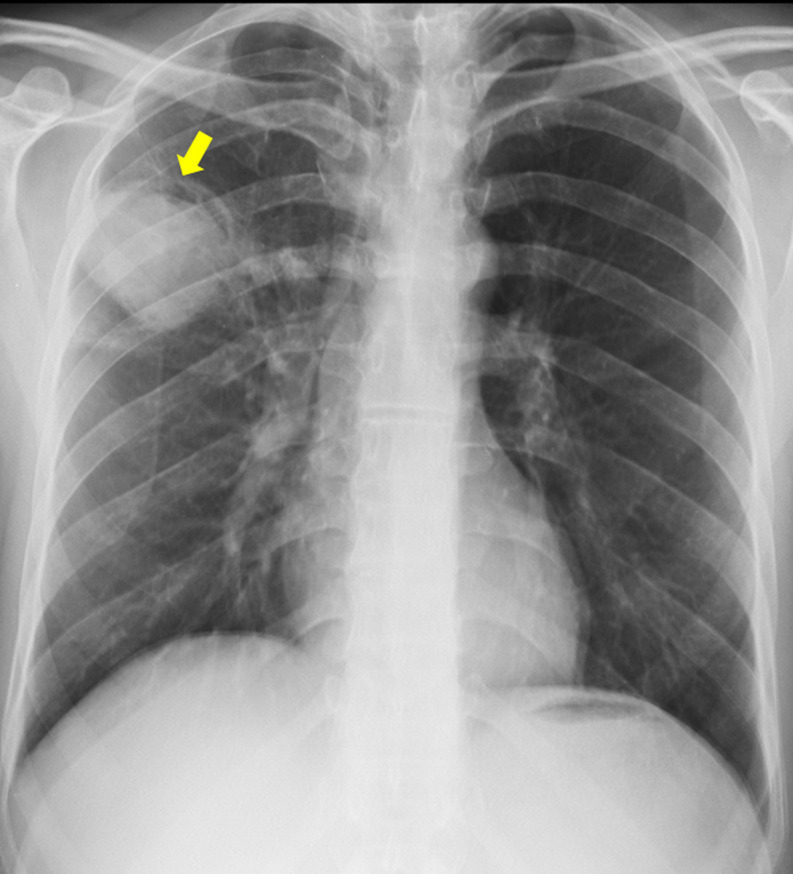
A 45-year-old male patient with a hydatid cyst in the right upper lobe showing a crescent sign (arrow) due to air entering between the pericyst and the laminar membrane.

**Figure 5.**
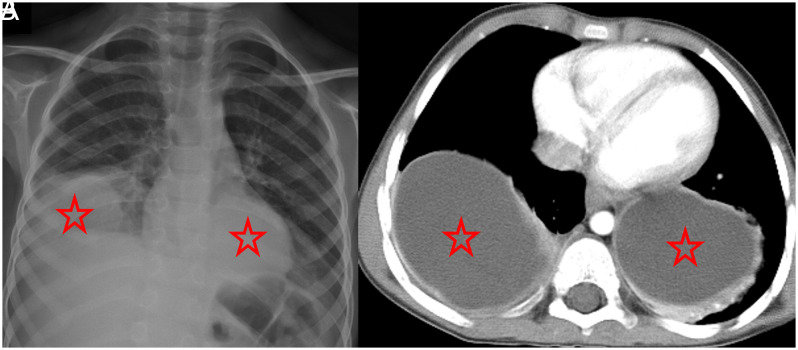
Posteroanterior chest radiograph (A) and axial chest CT (B) of a 5-year-old male patient with intact cysts measuring approximately 75 mm in the right lower lobe and 65 mm in the left lung (asterisks).

In regions where hydatid cysts are endemic, diagnosis is generally straightforward, especially in uncomplicated cases. Clinical and radiological evaluation is usually sufficient. In complicated cases, supporting radiological images with serology can be a useful research method for this disease. Leukocytosis, eosinophilia, and elevated erythrocyte sedimentation rate are nonspecific parameters observed in patients with this infection and are not diagnostic features.^14,15^ Radiologically, intact cysts present as well-defined, oval, and opaque lesions. When ruptured, they can result in various radiological appearances, such as the water lily sign, double-arch sign, dry cyst sign, air bubble sign, and serpent sign. These appearances can be mistaken for other pulmonary diseases, particularly lung cancer in adults (Figures 1-5).^6^

## Therapeutic Approach

Surgical intervention is the primary treatment for pulmonary hydatid cysts. Although medical treatment can be used, it is not recommended as extensively as it is for hepatic cysts. It is important to note that the PAIR (Puncture, Aspiration, Injection, Re-aspiration) method is not recommended for pulmonary hydatid cysts, unlike in hepatic cysts.^6^

## Surgical Treatment

The surgical treatment of hydatid cysts involves completely removing the cyst by taking out the inside, making sure it does not get contaminated or spill out, carefully closing the bronchial openings, dealing with any remaining spaces, and trying to keep as much of the lungs as possible intact. The surgical approach is dependent on a number of factors, including the size of the cyst, whether it is intact or ruptured, whether it is solitary or multiple, whether it is unilateral or bilateral, and the extent of lung parenchyma destruction.^16^

### Surgical Methods from Past to Present

Various techniques have been described for the surgical treatment of pulmonary hydatid cysts from the past to the present. Enucleation (Ugon method) is recommended for cysts smaller than 5 cm. This technique involves removing the intact germinal membrane along with the cyst. However, it is not recommended for larger cysts due to the risk of rupture and is rarely practiced. On the other hand, pericystectomy (Perez-Fontana method) involves removing the pericyst along with the hydatid cyst. It is not preferred nowadays due to its association with a high risk of air leak, tension pneumothorax, or bronchopleural fistula formation, particularly in delayed cases. The Figuera technique for open aspiration is similar to the method used for hepatic hydatid cysts. It involves PAIR. This procedure aspirates the cyst membranes and daughter vesicles. Although less invasive, this procedure still carries a risk of infection and air leak within the cavity, which can lead to the formation of empyema. This procedure is not widely accepted. Cystotomy with capitonnage, also known as Barrett’s method, involves cystotomy, aspiration of cyst fluid, and removal of the germinal membrane. The application of this technique serves to mitigate the likelihood of residual cavity infection, air leak, and empyema formation through the utilization of capitonnage. It is important to note that all commonly used techniques are fundamentally based on Barrett’s method. A cystotomy with the additional procedure of bronchial opening closuredoes not involve capitonnage, which results in less distortion of lung parenchyma. However, it does increase the risk of air leaks and infections. The Posadas methodis comparable to the Barrett method; however, in this approach, the bronchial airways are opened prior to the closure of the capitonnage. This reduces the likelihood of infection, air leak, and empyema formation within the residual cystic cavity. Therefore, the procedure’s outcome is satisfactory, except for causing atelectasis due to lung parenchymal distortion. The most frequently employed surgical technique in the treatment of pulmonary hydatid cysts is cystotomy and capitonnage, which is the Posadas method. However, in some cases, pulmonary resection may also be required.^14,17-20^

### Capitonnage and Uncapitonnage

Pulmonary hydatid cysts are universally agreed to be treated surgically. However, there is significant debate regarding whether or not to perform capitonnage. Some authors argue that after performing cystotomy and removing the membrane, it is sufficient to close the open bronchial openings.^21,22^ These studies suggest that capitonnage alone does not provide additional benefits compared to cystotomy and closure of the bronchial openings. However, some authors recommend the use of capitonnage, claiming that it offers additional benefits and is linked to better postoperative outcomes.^23-25^ The objective of capitonnage is to eliminate the residual cavity in order to prevent prolonged air leaks and postoperative abscess formation. This is achieved by applying a series of purse-string sutures from the base of the cyst wall until the cavity is completely closed. There are concerns, particularly with infected and complicated cysts, about the possibility of infection and tearing of lung tissue due to capitonnage sutures. As a result, the necessity of capitonnage is still a topic of debate. A recent systematic review and meta-analysis analyzed 12 studies comparing cases with and without capitonnage. The study found that overall complications were 3.8 times less, prolonged air leaks were 4.2 times less, and empyema was 4.8 times less in the group where capitonnage was performed compared to the group where it was not performed. Moreover, no notable discrepancies were observed between the 2 groups with respect to the incidence of atelectasis and the length of hospitalization. These results highlight the importance of capitonnage.^26^

### Bilateral Hydatid Cysts

In 85%-90% of cases, patients with echinococcosis exhibit single-organ involvement, with over 70% of them having a solitary cyst.^27^ Pulmonary hydatid cysts reportedly lead to bilateral lung involvement in 14% of cases.^1^ In cases where bilateral cysts are present, surgical planning should be based on a comprehensive evaluation of the intactness or complication status, diameters, and risk of dissemination of the cysts. Like many authors, we also recommend treating bilateral cysts with a 2-stage operation.^28-30^ If a patient has both an intact hydatid cyst in one lung and auptured complicated cyst in the other lung, the intact cyst should be given priority during surgery. Similarly, priority should be given to the hemithorax with the larger diameter cyst, the cyst located more centrally, or the hemithorax containing more cysts. These cases are typically approached thoracotomically or thoracoscopically, with a 2-4 week interval between surgeries. However, some surgeons argue that single-stage surgery is superior to the classical 2-stage operation because it reduces hospital stay, morbidity, and cost.^31,32^ Single-stage surgery involves simultaneous bilateral thoracotomy/video-assisted thoracoscopic surgery (VATS), median sternotomy, and Clamshell incision. One significant disadvantage of single-stage surgery is the risk of complications in both lungs simultaneously.

### Children and Adults

There is no difference in the surgical treatment of hydatid cysts between children and adults. However, due to the much faster growth of hydatid cysts in children compared to adults, it is recommended that surgical treatment be initiated as soon as possible when a hydatid cyst is detected in children.^11^ It is important to preserve the lung parenchyma as much as possible, especially in children, due to the rapid healing of lung tissue.

### Giant Cysts

**Figure 6.**
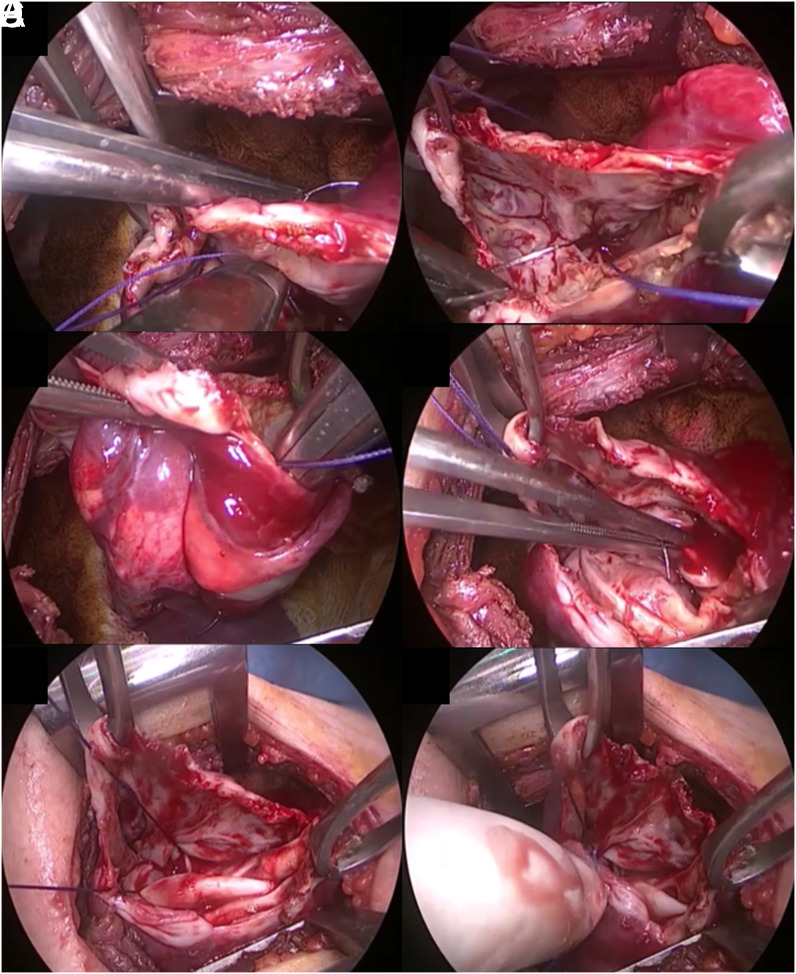
The modified capitonnage technique (Aydin technique) developed for the surgical treatment of a giant pulmonary hydatid cyst is illustrated. The suture entering the pericystic wall (A) is retrieved from the pleura (B) and the same procedure is applied to the opposite wall of the pericystic cavity (C, D). The sutures within the pericyst are ligated and cut (E, F).

Hydatid cysts are commonly referred to as giant cysts when they reach 10 cm in diameter or more. According to reports, giant cysts are more prevalent in the pediatric and young age groups than in adults.^33^ In some cases, patients with giant hydatid cysts may experience pleural complications, which may require major surgical procedures like decortication. Postoperative complications can be more anticipated in pulmonary giant hydatid cysts due to their complicated nature and increased bronchial associations compared to small cysts. Capitonnage is advantageous in cases of giant pulmonary hydatid cysts because of the presence of numerous bronchial openings. The modified capitonnage method, known as the Aydin method, allows for a more robust and secure application of capitonnage in giant and complicated hydatid cysts.^34^ In contrast to traditional purse-string capitonnage, which involves sutures passing from the inside of the pericyst wall and exiting through the intact pleura outside to prevent loosening and disruption due to inflammation, the modified method involves inserting sutures from a point near the lung parenchyma and re-entering the cavity from the pericyst wall (Figure 6). This technique is particularly useful for larger areas of the cavity, especially in cases where the pericyst wall is inflamed. It has been observed that this method is effective in preventing significant complications, such as postoperative empyema and prolonged air leakage.

### Lobectomy and Pneumonectomy

A lobectomy is recommended for hydatid cysts that involve more than 50% of a lobe, for infected cysts that are unresponsive to treatment, for cases of multiple cysts in a single lobe, and for cases with associated bronchiectasis, pulmonary fibrosis, or severe bleeding. Anatomical resection is also indicated when a complicated hydatid cyst cannot be distinguished from aspergilloma or lung cancer.^35^ However, it is recommended to consider parenchyma-preserving procedures primarily in children and young adults, regardless of the size of the cyst, as preservation of pulmonary parenchyma is a fundamental principle of hydatid cyst surgery. Nevertheless, cyst rupture can result in infection, parenchymal destruction, and abscess formation. In such cases, extensive wedge or lobectomy resection may be necessary.^36^

## Surgical Outcomes

Hydatid cyst surgery typically produces satisfactory outcomes. However, as with any surgical procedure, there are risks of complications and mortality. The postoperative complication rate associated with hydatid cyst disease has been reported to range from 1% to 39%, with a mortality rate of 0%-2%.^37-41^ The most common complication is a prolonged air leak. It has been documented that postoperative complications are more prevalent in ruptured and large-sized cysts. Intraoperative complications typically involve hydatid cyst rupture and bleeding. Postoperative complications may occur immediately or be delayed. Postoperative complications to consider include prolonged air leak, bronchopleural fistula, hemopneumothorax, empyema, and atelectasis.^37-41^

## Medical Therapy

Indications for medical treatment of pulmonary hydatid cysts include small cysts, high surgical risk, refusal of surgical treatment, multiorgan failure, multiple cysts, and patients with intraoperative spillage of hydatid fluid. Medical intervention is contraindicated in cases of large cysts at risk of rupture, calcified or inactive cysts, patients with bone marrow depression, and during pregnancy.^9,20,27,42-44^

Albendazole is the most preferred drug worldwide due to its better bioavailability, higher efficacy, and lower required dosage compared to mebendazole. The recommended dosage is 10-15 mg/kg/day, administered twice daily, and taking the drug with fat-rich meals can increase its bioavailability. The optimal duration of chemotherapy for pulmonary hydatid cysts is unknown, but it is recommended to start taking albendazole 15 days before surgery and continue for at least 3-6 months.^9,27,42,43^ The efficacy of preoperative or postoperative treatment for pulmonary hydatidosis has not been clearly studied. Traditionally, preoperative treatment has been recommended based on evidence from studies of liver hydatid cysts to prevent regrowth and postoperative intra-operative shedding. However, some authors, including ourselves, suggest that medical treatment with benzimidazoles should not be considered preoperatively.^20,42^ Rupture of a pulmonary hydatid cyst can result in asphyxia and anaphylaxis. Additionally, medical treatment may complicate the surgical procedure, necessitating surgical intervention. It is important to avoid rupturing the cyst during medical treatment.

Albendazole-induced hepatotoxicity is common. Monitoring of liver enzymes and complete blood count during treatment is mandatory, starting at weekly intervals at the start of treatment and continuing monthly and at longer intervals after stabilization.^42^ In the past, the drug was given in courses of several months, interrupted at 14-day intervals. These intervals were given to prevent hepatotoxicity. However, studies have shown that continuous treatment is more effective than cyclic treatment without an increase in side effects.^9^ The authors recommend albendazole treatment after surgery to prevent cyst rupture. In a study of 153 patients with pulmonary hydatid cysts who underwent 170 operations, albendazole at a dose of 15 mg/kg/day was administered postoperatively in 2 cycles of 15 days each. Patients were rested for 10 days in between, and complete blood counts and liver function tests were performed. In the case of bilateral cysts, albendazole treatment was started after the second operation. The occurrence of fever, malaise, and dizziness was observed in a single patient who had been treated with albendazole. A partial increase in liver enzymes was noted in 16 patients (10.5%) following treatment with albendazole. Only 1 patient experienced mild leucopenia and neutropenia. One patient underwent a second operation after 30 months due to recurrence. Albendazole treatment did not need to be discontinued in any of the patients. This study demonstrated the efficacy of a shorter course of albendazole prophylaxis after surgery in preventing recurrences.^44^

## Conclusion

The diagnosis of intact pulmonary hydatid cysts is generally straightforward, especially in endemic regions. Surgery is the primary treatment for pulmonary hydatid cysts. Surgical treatment should be started as soon as possible after diagnosis, especially in children, because of the shorter doubling time. In the case of bilateral pulmonary hydatid cysts, sequential thoracotomies 2-4 weeks apart, prioritizing the hemithorax for centrally located, multiple, intact, and larger cysts, may be effective. For giant hydatid cysts, the modified capitonnage technique may reduce complication rates. The procedure of cystotomy and capitonnage significantly reduces postoperative complications compared to cases where capitonnage is not performed. In cases of intact pulmonary hydatid cysts, albendazole treatment should be given postoperatively to prevent cyst rupture, asphyxia, and anaphylaxis.

## Data Availability

The data that support the findings of this study are available on request from the corresponding author.

## References

[1] Aydin Y Ulas AB Ince I , et al. Large case series analysis of cystic echinococcosis. Indian J Surg. 2021;83(suppl 4):897 906. (10.1007/s12262-021-03061-0)

[2] Mfingwana L Goussard P van Wyk L , et al. Pulmonary Echinococcus in children: a descriptive study in a LMIC. Pediatr Pulmonol. 2022;57(5):1173 1179. (10.1002/ppul.25854) 35122423

[3] Moro P Schantz PM . Cystic echinococcosis in the Americas. Parasitol Int. 2006;55(suppl):S181 S186. (10.1016/j.parint.2005.11.048) 16376604

[4] Acosta-Jamett G Hernández FA Castro N , et al. Prevalence rate and risk factors of human cystic echinococcosis: a cross-sectional, community-based, abdominal ultrasound study in rural and urban north-central Chile. PLoS Negl Trop Dis. 2022;16(3):e0010280. (10.1371/journal.pntd.0010280) 35263331 PMC8936472

[5] Tamarozzi F Akhan O Cretu CM , et al. Epidemiological factors associated with human cystic echinococcosis: a semi-structured questionnaire from a large population-based ultrasound cross-sectional study in eastern Europe and Turkey. Parasit Vectors. 2019;12(1):371. (10.1186/s13071-019-3634-1) 31358039 PMC6664724

[6] Aydin Y Ulas AB Ahmed AG Eroglu A . Pulmonary hydatid cyst in children and adults: diagnosis and management. Eurasian J Med. 2022;54(suppl 1):133 140. (10.5152/eurasianjmed.2022.22289) PMC11163342 36655457

[7] Santucciu C Bonelli P Peruzzu A , et al. Cystic echinococcosis: clinical, immunological, and biomolecular evaluation of patients from Sardinia (Italy). Pathogens. 2020;9(11):907. (10.3390/pathogens9110907) 33143032 PMC7693143

[8] Romig T Deplazes P Jenkins D , et al. Ecology and life cycle patterns of Echinococcus species. Adv Parasitol. 2017;95:213 314. (10.1016/bs.apar.2016.11.002) 28131364

[9] Sarkar M Pathania R Jhobta A Thakur BR Chopra R . Cystic pulmonary hydatidosis. Lung India. 2016;33(2):179 191. (10.4103/0970-2113.177449) 27051107 PMC4797438

[10] Ben Abdallah AK Zouari M Haj Mansour M , et al. Hydatid cyst of the lung in children: a diagnosis not to be missed. Iran J Public Health. 2019;48(4):767 769. (10.18502/ijph.v48i4.1012) 31110989 PMC6500519

[11] Aydın Y Özgökçe M Bilal Ulas AB , et al. Doubling time in pulmonary and hepatic hydatid cysts. Turk Gogus Kalp Damar Cerrahisi Derg. 2024;32(2):185 194. (10.5606/tgkdc.dergisi.2024.25844) 38933308 PMC11197416

[12] Aydın Y Çelik M Ulaş AB Eroğlu A . Transdiaphragmatic approach to liver and lung hydatid cysts. Turk J Med Sci. 2012;42(suppl 2):1388 1393. (10.3906/sag-1204-22)

[13] Aydin Y Dostbil A Araz O , et al. Pre-school children with hydatid lung disease. Acta Chir Belg. 2013;113(5):340 345. (10.1080/00015458.2013.11680941) 24294798

[14] Rawat S Kumar R Raja J Singh RS Thingnam SKS . Pulmonary hydatid cyst: review of literature. J Fam Med Prim Care. 2019;8(9):2774 2778. (10.4103/jfmpc.jfmpc_624_19) PMC6820383 31681642

[15] Aydin Y Altuntas B Kaya A Ulas AB Uyanık MH Eroglu A . The availability of Echinococcus IgG ELISA for diagnosing pulmonary hydatid cysts. Eurasian J Med. 2018;50(3):144 147. (10.5152/eurasianjmed.2018.16104) 30515031 PMC6263238

[16] Thapa B Sapkota R Kim M Barnett SA Sayami P . Surgery for parasitic lung infestations: roles in diagnosis and treatment. J Thorac Dis. 2018;10(suppl 28):S3446 S3457. (10.21037/jtd.2018.08.32) 30505532 PMC6218367

[17] Nabi MS Waseem T . Pulmonary hydatid disease: what is the optimal surgical strategy? Int J Surg. 2010;8(8):612 616. (10.1016/j.ijsu.2010.08.002) 20732461

[18] Kiliccalan I Cingoz OT . Pulmonary hydatid cyst: pathophysiology, etiopathogenesis, diagnosis and treatment. Maltepe Tıp Derg. 2023;15(3):105 109. (10.35514/mtd.2024.102)

[19] Burgos R Varela A Castedo E , et al. Pulmonary hydatidosis: surgical treatment and follow-up of 240 cases. Eur J Cardiothorac Surg. 1999;16(6):628 634. (10.1016/s1010-7940(99)00304-8) 10647832

[20] Lupia T Corcione S Guerrera F , et al. Pulmonary echinococcosis or lung hydatidosis: a narrative review. Surg Infect (Larchmt). 2021;22(5):485 495. (10.1089/sur.2020.197) 33297827

[21] Eren MN Balci AE Eren S . Non-capitonnage method for surgical treatment of lung hydatid cysts. Asian Cardiovasc Thorac Ann. 2005;13(1):20 23. (10.1177/021849230501300105) 15793045

[22] Turna A Yilmaz MA Haciibrahimoğlu G Kutlu CA Bedirhan MA . Surgical treatment of pulmonary hydatid cysts: is capitonnage necessary? Ann Thorac Surg. 2002;74(1):191 195. (10.1016/s0003-4975(02)03643-3) 12118757

[23] Kosar A Orki A Haciibrahimoglu G Kiral H Arman B . Effect of capitonnage and cystotomy on outcome of childhood pulmonary hydatid cysts. J Thorac Cardiovasc Surg. 2006;132(3):560 564. (10.1016/j.jtcvs.2006.05.032) 16935111

[24] Nabi MS Waseem T Tarif N Chima KK . Pulmonary hydatid disease: is capitonnage mandatory following cystotomy? Int J Surg. 2010;8(5):373 376. (10.1016/j.ijsu.2010.05.007) 20681056

[25] Bilgin M Oguzkaya F Akçali Y . Is capitonnage unnecessary in the surgery of intact pulmonary hydatic cyst? ANZ J Surg. 2004;74(1-2):40 42. (10.1046/j.1445-1433.2003.02684.x) 14725704

[26] Aydın Y Kasalı K Ulaş AB Dostbil A İnce İ Eroglu A . Comparing capitonnage and Uncapitonnage techniques for pulmonary hydatid cysts: a systematic review and meta-analysis. Eurasian J Med. 2023;55(1 suppl 1):S35 S42. (10.5152/eurasianjmed.2023.22281) 37916996 PMC11075026

[27] Morar R Feldman C . Pulmonary echinococcosis. Eur Respir J. 2003;21(6):1069 1077. (10.1183/09031936.03.00108403) 12797504

[28] Önal Ö Demir ÖF . Is bilateral staged muscle-sparing thoracotomy performed within 1 week for lung hydatid cysts safe for pediatric patients? Turk Thorac J. 2018;19(2):84 88. (10.5152/TurkThoracJ.2018.17039) 29755812 PMC5937815

[29] Hasdıraz L Onal O Oguzkaya F . Bilateral staged thoracotomy for multiple lung hydatidosis. J Cardiothorac Surg. 2013;8:121. (10.1186/1749-8090-8-121) 23641938 PMC3668183

[30] Aydin Y Ulas AB Kasali K , et al. Treatment approach in bilateral pulmonary hydatid cysts: analysis of 107 consecutive cases. Indian J Thorac Cardiovasc Surg. 2024;40(6):669 674. (10.1007/s12055-024-01750-5) 39416332 PMC11473502

[31] Lone GN Bhat MA Ali N Bashir A Garcoo SA . Single-stage bilateral minimally invasive approach for pulmonary hydatid disease: an alternative technique. J Thorac Cardiovasc Surg. 2002;124(5):1021 1024. (10.1067/mtc.2002.122315) 12407388

[32] Atalay A Salih OK Gezer S , et al. Simultaneous heart and bilateral lung hydatid cyst operated in a single session. Heart Lung Circ. 2013;22(8):682 684. (10.1016/j.hlc.2012.11.014) 23265691

[33] Usluer O Ceylan KC Kaya S Sevinc S Gursoy S . Surgical management of pulmonary hydatid cysts: is size an important prognostic indicator? Tex Heart Inst J. 2010;37(4):429 434. 20844615 PMC2929855

[34] Aydin Y Ulas AB Ince I , et al. Modified capitonnage technique for giant pulmonary hydatid cyst surgery. Interact Cardiovasc Thorac Surg. 2021;33(5):721 726. (10.1093/icvts/ivab152) 34041544 PMC8932517

[35] Nistor CE Berbece S Batog O Ciuche A . Pulmonary hydatidosis - an abandoned surgical approach. Chirurgia (Bucur). 2020;115(3):394 403. (10.21614//chirurgia.115.3.394) 32614296

[36] Onal O Demir OF . Is anatomic lung resection necessary in surgical treatment of giant lung hydatid cysts in childhood? Ann Thorac Cardiovasc Surg. 2017;23(6):286 290. (10.5761/atcs.oa.17-00023) 28883209 PMC5738449

[37] Yaldiz S Gursoy S Ucvet A Yaldiz D Kaya S . Capitonnage results in low postoperative morbidity in the surgical treatment of pulmonary echinococcosis. Ann Thorac Surg. 2012;93(3):962 966. (10.1016/j.athoracsur.2011.11.011) 22265202

[38] Ksia A Fredj MB Zouaoui A , et al. Capitonnage seems better in childhood pulmonary hydatid cyst surgery. J Pediatr Surg. 2020;55(4):752 755. (10.1016/j.jpedsurg.2019.05.009) 31138449

[39] Ahmadinejad M Hashemi M Azizallahi N . Evaluation of prognostic factors associated with postoperative complications following pulmonary hydatid cyst surgery. Open Respir Med J. 2020;14:16 21. (10.2174/1874306402014010016) 32742527 PMC7372731

[40] Bagheri R Haghi SZ Amini M Fattahi AS Noorshafiee S . Pulmonary hydatid cyst: analysis of 1024 cases. Gen Thorac Cardiovasc Surg. 2011;59(2):105 109. (10.1007/s11748-010-0690-z) 21308436

[41] Dogru MV Sezen CB Aker C , et al. Evaluating giant hydatid cysts: factors affecting mortality and morbidity. Ann Thorac Cardiovasc Surg. 2021;27(3):164 168. (10.5761/atcs.oa.20-00178) 33162437 PMC8343032

[42] Weber TF Junghanss T Stojković M . Pulmonary cystic echinococcosis. Curr Opin Infect Dis. 2023;36(5):318 325. (10.1097/QCO.0000000000000962) 37578473 PMC10487362

[43] Fattahi Masoom SH Lari SM Fattahi AS Ahmadnia N Rajabi M NaderiKalat M . Albendazole therapy in human lung and liver hydatid cysts: a 13-year experience. Clin Respir J. 2018;12(3):1076 1083. (10.1111/crj.12630) 28319358

[44] Aydin Y Ulas AB Ince I , et al. Evaluation of albendazole efficiency and complications in patients with pulmonary hydatid cyst. Interact Cardiovasc Thorac Surg. 2022;34(2):245 249. (10.1093/icvts/ivab259) 34587626 PMC8766210

